# Highly selective palladium–benzothiazole carbene-catalyzed allylation of active methylene compounds under neutral conditions

**DOI:** 10.3762/bjoc.11.111

**Published:** 2015-06-10

**Authors:** Antonio Monopoli, Pietro Cotugno, Carlo Giorgio Zambonin, Francesco Ciminale, Angelo Nacci

**Affiliations:** 1 Department of Chemistry, University of Bari Via Orabona 4, 70126 Bari, Italy; 2 CNR-ICCOM, Department of Chemistry, University of Bari, Via Orabona 4, 70126 Bari, Italy

**Keywords:** active methylene compounds, allylic carbonates, Pd–benzothiazol-2-ylidene complex, Tsuji–Trost allylation

## Abstract

The Pd–benzothiazol-2-ylidene complex **I** was found to be a chemoselective catalyst for the Tsuji–Trost allylation of active methylene compounds carried out under neutral conditions and using carbonates as allylating agents. The proposed protocol consists in a simplified procedure adopting an in situ prepared catalyst from Pd_2_dba_3_ and 3-methylbenzothiazolium salt **V** as precursors. A comparison of the performance of benzothiazole carbene with phosphanes and an analogous imidazolium carbene ligand is also proposed.

## Introduction

The α-allylation of carbonyl compounds is one of the most important reactions in organic chemistry, since it opens the way to the synthesis of a plethora of interesting molecules such as pheromones, perfumes, or bio-active compounds such as prostaglandin E_2_ or F_2α_. After the pioneering works by Tsuji [1–2] and Trost [3–4], the Pd-catalyzed allylation of various nucleophiles is a largely used strategy and a variety of efficient and robust homogeneous [5–9] and heterogeneous [10–13] Pd catalysts have been reported, until now. Recently, synergistic or cooperative catalysis has been also described for the Tsuji–Trost allylation, in which the use of a base in combination with a Pd species resulted in better outcomes [14–21].

However, some of these protocols suffer for severe drawbacks such as long reaction times [18,22], undesirable overreactions giving the diallylated compounds [23–24], the need for catalysts that are tedious to prepare [18–19], and the use of toxic or expensive ligands such as phosphanes or phosphites [3,25]. Therefore, the careful selection of a suitable ligand capable for replacing phosphines and improving palladium activity is still mandatory in these kind of reactions. Among various ligands, N-heterocyclic carbenes (NHCs) have gained greater importance in organometallic chemistry. Unlike phosphanes, NHCs are not toxic and insensitive to air, heat and moisture.

Moreover, the introduction of substituents onto the heterocyclic ring enables the tuning of their steric and electronic properties affecting the catalytic activity of the resulting metal complex [26–29]. Although NHC–Pd complexes have been employed in many C–C bond-forming reactions, to the best of our knowledge they have been scarcely applied to the allylic alkylation of nucleophiles [30–36].

**Figure 1:**
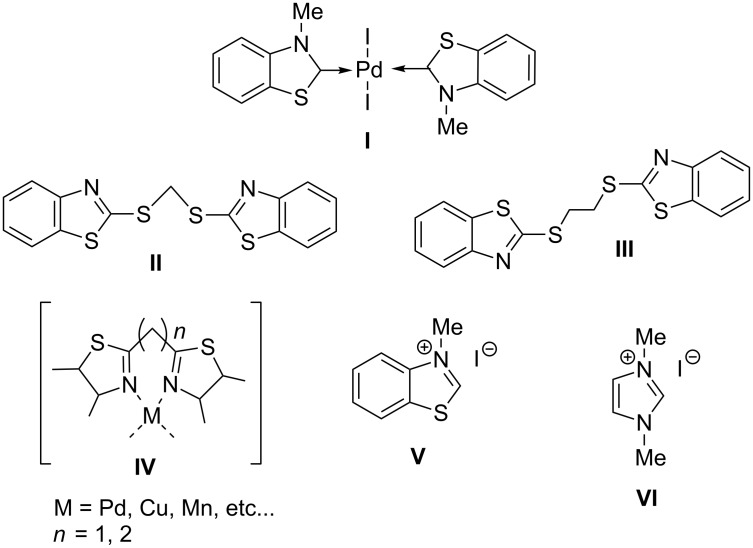
Complexes and ligands employed.

Some years ago, we synthesized the first example of Pd–benzothiazol-2-ylidene complex **I** (Figure 1), which proved to be an efficient catalyst for several C–C coupling reactions (like carbonylations and Heck olefinations) carried out in both conventional solvents [37] and in ionic liquids [38]. Complex **I** is easily prepared from the corresponding thiazolium salt **V** and Pd(OAc)_2_, and due to its high stability the complex can be purified by silica gel chromatography.

**Scheme 1:**
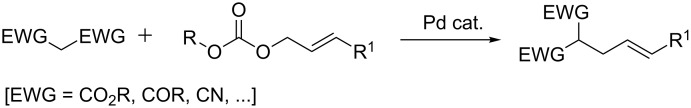
Pd-catalyzed α-allylation of active methylene compounds.

We report here the use of dicarbenediiodopalladium(II) complex **I**, prepared in situ, as a precatalyst in the Tsuji–Trost allylation of active methylene compounds using carbonates as allylating agents (Scheme 1).

## Results and Discussion

Allylic carbonates are suitable reagents for the Tsuji–Trost allylation as they enable to work under neutral conditions. The base required for methylene deprotonation, the alkoxide anion (RO^−^), can in fact originate (in situ) from the degradation of the allylic carbonate by the Pd catalyst [2]. Optimisation of the reaction conditions was carried out on the model substrate diethyl malonate by varying several parameters such as ligands, Pd sources, catalyst loading, temperature and solvents (Table 1).

**Table 1:** Optimization of reaction conditions.^a^

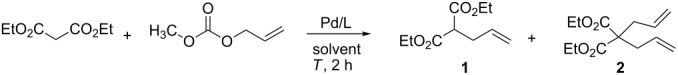							

Entry	Pd source (%)	Ligand (mol %)		Solvent	*T* (°C)	Conv. %^b^	Selectivity^c^ **1**:**2**

		PPh_3_	other				

1	Pd(OAc)_2_ (2)	4	**II** (4)	CH_2_Cl_2_	25	100	70:30
2	Pd(OAc)_2_ (2)	–	**II** (20)	CH_2_Cl_2_	25	<5	–
3	Pd(OAc)_2_ (2)	20	–	CH_2_Cl_2_	25	100	55:45
4	Pd_2_dba_3_ (2)	4	**II** (4)	CH_2_Cl_2_	25	100	60:40
5	Pd(OAc)_2_ (2)	4	**II** (4)	THF	25	10	99:1
6	Pd(OAc)_2_ (2)	20	–	THF	70	100	50:50
7	Pd_2_dba_3_ (2)	4	**II** (4)	THF	70	100	63:37
8	Pd_2_dba_3_ (5)	10	**II** (10)	THF	70	100	70:30
9	Pd(OAc)_2_ (2)	4	**III** (4)	CH_2_Cl_2_	25	100	65:35
10	Pd_2_dba_3_ (2)	4	**III** (4)	CH_2_Cl_2_	25	100	62:38
11	Pd_2_dba_3_ (2)	–	**III** (20)	CH_2_Cl_2_	25	<5	–
**12**	**I (2)**	–	–	**THF**	**25**	**100**	**99:1**
13	Pd_2_dba_3_ (2)	–	**V** (4)^d^	THF	25	48	96:4
**14**	**Pd_2_dba_3 _(2)**	–	**V (8)**^d^	**THF**	**25**	**100**	**97:3**
15	Pd(OAc)_2_ (2)	–	**V** (8)^d^	THF	25	100	95:5
16	Pd_2_dba_3_ (2)	–	**VI** (8)^d^	THF	25	16	98:2

^a^Reaction conditions: diethyl malonate (1.2 mmol), methyl allyl carbonate (1 mmol), Pd source, ligand, in 5 mL of solvent. ^b^Conversions are evaluated based on disappearance of carbonate. ^c^The **1**:**2** ratio is determined on the base of GLC peak areas. ^d^NaH was added to generate carbene ligand (see Experimental section).

With regard to the influence of ligands, the efficiency of benzothiazole–carbene was compared with that of sulfides **II** and **III**, both in the presence and in the absence of PPh_3_, as it is well known that also chelating *N*-heterocyclic ligands bearing methylene or ethylene bridges can form very active catalysts in these kind of reactions (structure **IV**) [39].

Nevertheless, from the data in Table 1 it clearly emerges that chelating *N*-ligands **II** and **III** were unproductive when used alone, giving very low conversion values (<5%, Table 1, entries 2 and 11). In contrast, PPh_3_ provided complete conversions but afforded almost equimolar mixtures of mono- and bis-allylated products (ca. 50:50, Table 1, entries 3 and 6). Disappointing results in terms of selectivity were also found by combining the two types of ligands, although in these cases a slightly higher selectivity in favour of mono-allylated product **1** was observed (on average 65:35) probably due to the steric influence of chelating ligands **II** and **III** (Table 1, entries 1, 4, 5, and 7–10). Other parameters such as temperature, solvents, catalyst loading and palladium sources proved to have a negligible effect on the reaction outcome.

A special behaviour was observed with Pd–carbene complex **I** as precatalyst. Indeed, with 2 mol % of **I** prepared ex situ [37], the reaction carried out in dry THF reached a complete conversion in only 2 hours at room temperature, with the selective formation of the mono-allylated product **1** (Table 1, entry 12). Interestingly, the same result was achieved using the Pd–carbene complex prepared in situ from Pd_2_dba_3_ and 3-methylbenzothiazolium iodide (**V**) as a carbene precursor (Table 1, entry 14). In this case, to generate the carbene ligand, the addition of the base NaH was necessary for the proton abstraction at the C2 position of the thiazolium salt. In addition, the use of sub-stoichiometric amounts of **V** afforded correspondingly lower conversions without altering the chemoselectivity of the process (Table 1, entry 13).

If palladium acetate was used as precatalyst, an induction period was observed, thus indicating the need for the reduction of the Pd(II) precatalyst to the Pd(0) active species (Table 1, entry 15).

Finally, we also compared the performance of our 3-methylbenzothiazol-2-ylidene carbene ligand with that of the analogous 1,3-dimethylimidazol-2-ylidene by Herrmann et al. [40], prepared in situ from 1,3-dimethylimidazolium iodide (**VI**).

The reaction carried out under the protocol conditions afforded in 2 h the monoallylated compound **1** in a low yield (16%) confirming the superior efficiency of benzothiazole–carbene (Table 1, entry 16).

A possible explanation of the different behavior displayed by these two carbene ligands can be found in the different aromatic character of their (benzothiazole and imidazole) heterocyclic rings. It is well known that this feature can strongly affect the nucleophilicity of these NHC species, and ultimately can influence their ability of acting as a σ-donor towards the metal. In particular, the higher the aromatic character of the heterocycle the lower is the back-donation by palladium, and this effect would enhance the electron density on the metal rendering it more reactive. On these bases, we can speculate that the benzothiazole–carbene ligand deriving from **V**, should be more active (being a better σ-donor) than the corresponding imidazole (deriving from **VI**) due to its higher aromatic character. Studies are in progress to verify this assumption.

With optimized conditions in hand, we widened the scope of our investigation by extending the coupling to a series of 1,3-dicarbonyl compounds and allylic carbonates (Table 2). For these reactions, to further simplify the operating procedure of the proposed protocol, we chose to use the palladium–carbene catalyst prepared in situ as reported above (Table 1, entries 20–22).

**Table 2:** α-allylation of 1,3-dicarbonyl compounds^a^.

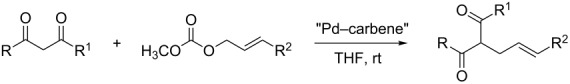						

Entry	Substrate	Allylic carbonate	*t* (h)	Product	Conv. (%)^b^	Yield (%)^b^

1	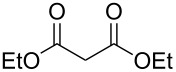	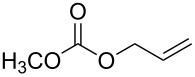	2	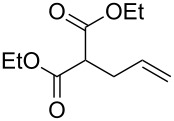 **1**	99	97
2		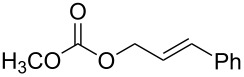	2	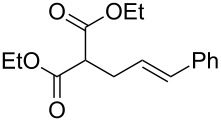 **2**	89	83^c^ (88)^b^
3		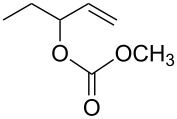	6	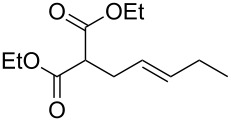 **3**	90	86^c^ (89)^b^

4	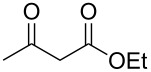	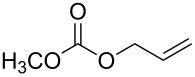	3	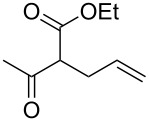 **4**	>99	98
5		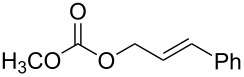	6	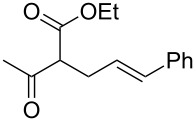 **5**	91	90
6		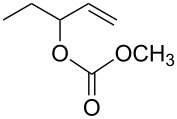	8	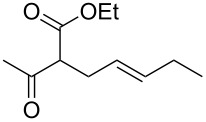 **6**	84	75^c^ (81)^b^

7	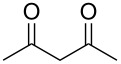	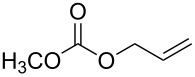	7	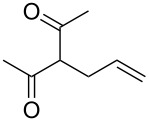 **7**	>99	64^d^
8		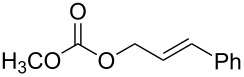	8	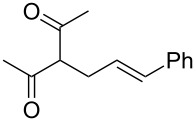 **8**	88	82
9		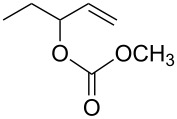	9	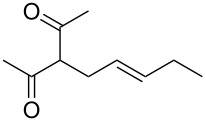 **9**	78	71^c^ (74)^b^

^a^Reaction conditions: dicarbonyl compound (1.2 mmol), allyl carbonate (1 mmol), Pd_2_dba_3_, (0.02 mmol) benzothiazolium iodide **V** (0.08 mmol), NaH (0.1 mmol) in 5 mL of solvent (see Supporting Information File 1). ^b^Conversions and yields were evaluated via GLC by using diethylene glycol di-*n*-butyl ether as internal standard. ^c^Isolated product. ^d^A mixture of mono- and diallylated products in a 64:36 ratio was formed.

The data in Table 2 show that the reactions proceeded smoothly with yields ranging from 64% to 98% and a complete selectivity in favour of the mono-allylated compounds in most of the examined combinations.

Predictably, the reactivity of β-dicarbonyl compounds (i.e., conversion values and reaction times) was found to depend on the nucleophilic strength of the intermediate enolates, and ultimately on p*K*_a_ values (reactivity scale: diethyl malonate p*K*_a_ 13.5 > acetoacetate p*K*_a_ 11.0 > acetylacetone p*K*_a_ 8.9). As an example, diethyl malonate reacted with allyl methyl carbonate much faster than acetylacetone, reaching the complete conversion in only 2 hours (vs 7 hours of acetylacetone, Table 2, entries 1 and 7).

In a similar predictable manner, selectivity was affected by the steric hindrance of allyl carbonate. This influence was evident in the case of the slower reactions of acetylacetone, for which the sterically more hindered cinnamyl- and pentenyl methyl carbonates afforded exclusively the monoallylated products (Table 2, entries 8 and 9), while the less hindered methyl allyl carbonate, afforded also remarkable amounts of the diallylated compounds (64:36 ratio, Table 2, entry 7).

## Conclusion

In conclusion, we have found that Pd-benzothiazole carbene complex **I** can act as an efficient catalyst for the Tsuji–Trost α-allylation of active methylene compounds carried out under neutral conditions and using carbonates as allylating agents. The proposed protocol is not only highly chemoselective, but occurs under very mild temperature conditions and the operating procedure is further simplified employing the catalyst prepared in situ. In addition, benzothiazol-2-ylidene ligands proved to be not only more efficient in terms of selectivity than the toxic phosphanes but can compete favourably also with the analogous and more widely used NHC carbenes deriving from imidazole.

## Supporting Information

File 1 General methods, synthetic procedures, characterization data of all new compounds.
