# Supplementary material for: Highly selective palladium–benzothiazole carbene-catalyzed allylation of active methylene compounds under neutral conditions
Source: Beilstein J Org Chem. 2015 Jun 10;11:994–9. doi: 10.3762/bjoc.11.111 (PMC4505175; doi:10.3762/bjoc.11.111)
Supplement: File 1 — General methods, synthetic procedures, characterization data of all new compounds. [file Beilstein_J_Org_Chem-11-994-s001.pdf]

Supporting Information

for

Highly selective palladium–benzothiazole carbene-catalyzed allylation of active methylene compounds under neutral conditions

Antonio Monopoli*[1], Pietro Cotugno[1], Carlo G. Zambonin[1], Francesco Ciminale[1] and Angelo Nacci*[1,2]

Address: [1]Department of Chemistry, University of Bari Via Orabona 4, 70126 Bari, Italy and [2]CNR-ICCOM, Department of Chemistry, University of Bari, Via Orabona 4, 70126 Bari, Italy

Email: Antonio Monopoli - antonio.monopoli@uniba.it; Angelo Nacci - angelo.nacci@uniba.it

*Corresponding authors

In memory of Dr. Francesco Paolo Monopoli

General methods, synthetic procedures, characterization data of all new compounds

Experimental

THF was distilled on sodium/benzophenone, while CH_2Cl_2 was purified by distillation on P_2O_5. Dicarbonyl substrates and ligands **II** and **III** are commercially available (Aldrich) and were used as received. Allylic carbonates were synthesized according to known procedures [1]. Allylation products **1**–**9** were identified by comparison of their spectral data (MS and ^{1}H NMR) with those reported in the literature. MS spectra were recorded on SHIMADZU QP-5000, while NMR spectra were recorded on Bruker AM 500 and Varian 200 machines with $CDCl_3$ as the solvent. Carbene

ligand precursors namely 3-methylbenzothiazolium iodide (**V**) [2a] and 1,3-dimethylimidazolium iodide (**VI**) [2b], together with complex **I** [2a] were prepared according to known procedures.

General procedure for the in situ allylation reaction. In a three-necked flask, 0.1 equiv of NaH (60%) and 0.08 mmol of *N*-methylbenzothiazolium iodide were refluxed in 5 mL of dry THF under inert atmosphere. After 30 minutes, the solution is cooled at room temperature, then Pd_2dba_3 is added and, after 2 minutes, 1.2 equiv of dicarbonyl compound and 1 equiv of allylcarbonate dissolved in 2 mL of THF, were added. The reaction was monitored by GC–MS. After reaction completion, the THF was removed under vacuum, the residue washed with water and extracted with ethyl acetate (3 × 5mL). Organic phases were collected, dried with sodium sulfate and evaporated under reduced pressure. Products were purified by silica gel chromatography (eluent: hexane/ethyl acetate in a proper ratio). Since all the obtained products are known, identification was accomplished by comparison of their spectral data (MS and ^{1}H NMR) with those reported in the literature.

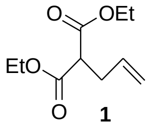

1

2-Allylmalonic acid diethyl ester (**1**)

GC-MS m/e (%): 200 (M^+, 0.6); 98 (b.p.); 127 (65); 109 (86) (lit. [3]).

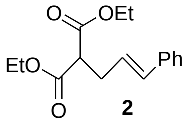

2

(*E*)-2-(3-Phenylallyl)malonic acid diethyl ester (**2**)

This product has been isolated by silica gel column chromatography (yellow oil, 83% of yield): ^{1}H-NMR (500 MHz): δ= 1.23 (6H, t, J=7.0 Hz, CH_3 ethyl group); 2.79 (2H, td like, J=7.4 and 1.4 Hz, CH_2 allyl group); 3.48 [1H, t, J=7.4 Hz, $C\underline{H}(CO_2Et)_2$]; 4.18 (4H, q, J=7.0 Hz, CH_2 ethyl group); 6.13 (1H, dt, J=15.8 and 7.2 Hz, PhCH=C$\underline{H}$); 6.45 (1H, d, J=15.8 Hz, PhC$\underline{H}$=CH); 7.16-7.39 (5H, m, aromatic protons). (lit. [4])

GC-MS m/e (%): 276 (M^+, 17); 129 (b.p.); 117 (34); 202 (25); 77 (6).

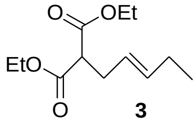

3

(*E*)-2-(Pent-2-en-1-yl)malonic acid diethyl ester (**3**).

This product has been isolated by silica gel column chromatography and identified by NMR. Yield of 98%. ^{1}H-NMR (200 MHz): δ= 0.98 (3H, t, J= 7.2 Hz, CH_3 allyl group); 1.28 (6H, t, J= 7.0 Hz, CH_3 ethoxyl group); 2.53 (2H, m, CH2 ethyl); 2.60 (2H, m, CH_2 allylic); 3.24 ([1H, t, J= 7.4, $C\underline{H}(CO_2Et)_2$]; 4.15 (4H, q, J= 7.0 Hz, CH_2 ethoxyl group); 5.45 (2H, m, olefinic protons). (lit. [5]).

GC-MS m/e (%): 228 (M^+, 3); 125 (b.p.); 41 (92); 97 (49); 81 (53); 55 (49).

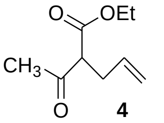

4

2-Acetyl-pent-4-enoic acid ethyl ester (**4**)

GC-MS m/e (%): 170 (M^+, 0.2); 43 (b.p.); 55 (23); 127 (28). (lit. [3])

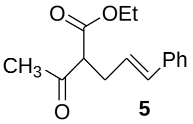

5

(*E*)-2-Acetyl-5-phenyl-pent-4-enoic acid ethyl ester (**5**)

GC-MS m/e (%): 246 (M^+, 12); 43 (b.p.); 157 (74); 203 (22), 77 (8). (lit. [6])

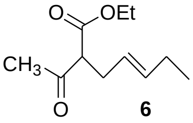

6

(*E*)-2-Acetyl-4-heptenoic acid ethyl ester (**6**)

This product has been isolated by silica gel column chromatography and identified by NMR. Yield of 87%. ^{1}H-NMR (200 MHz): δ= 0.90 (3H, t, J=7.0 Hz, CH_3 ethyl); 1.28 (3H, t, J=7.1 Hz, CH_3 ethoxyl group); 1.90-2.00 (2H, m, CH_2 ethyl); 2.20 (3H, s, CH3CO); 2.40-2.50 (2H, m, CH_2 allylic); 3.45 [1 H, t, J= 7.2, C$\underline{H}$$(CO)_2$]; 4.15 (2H, q, J= 7.1 Hz, CH_2 ethoxy group); 5.20-5.30 (1H, m, olefinic proton); 5.50-5.60 (1H, m, olefinic proton). (lit. [7]).

GC-MS m/e (%): 198 (M^+, 0.4); 43 (b.p.); 41 (23); 155 (17); 109 (15); 81 (14).

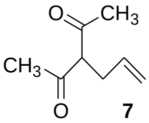

7

3-Allylpentan-2,4-dione (**7**)

GC-MS m/e (%): 140 (M^+, 0.3); 43 (b.p.); 97 (30). (lit. [8])

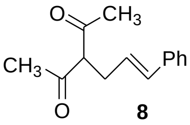

8

(*E*)-3-(3-Phenylallyl)-pentan-2,4-dione (**8**)

GC-MS m/e (%): 216 (M^+, 0.7); 43 (b.p.); 173 (30); 91 (45); 77 (3). (lit. [6])

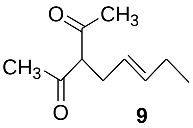

9

(*E*)-3-(Pent-2-enyl)pentan-2,4-dione (**9**)

This product has been isolated by silica gel column chromatography and identified by NMR. Yield of 91%. ^{1}H-NMR (500 MHz): δ= 0.90 (3H, t, J=7.0 Hz, CH_3 ethyl); 1.85-1.95 (2H, m, CH_2 ethyl); 2.05 (6H, s, CH_3CO); 2.52 (2H, td like, J= 8.2 and 1.3 Hz, CH_2 allylic); 3.67 [1H, t, J= 7.9, $CH(COMe)_2$]; 5.22-5.29 (1H, m, olefinic proton. Irradiating on δ= 2.52 we obtained a doublet of triplet, J= 14.1 and J= 1.70 Hz, indicating a *trans* isomery); 5.48-5.56 (1H, m, olefinic proton). (lit. [9]).

GC-MS m/e (%): 168 (M^+, 0.1); 43 (b.p.); 125 (17); 41 (11).

References

1 Tsuji, J.; Shimizu, I.; Minami, I.; Ohashi, Y.; Sugiura, T.; Takahashi, k. *J. Org. Chem.* **1985**, *50*, 1523-1529.

2 a) Calo´, V.; Del Sole, R.; Nacci, A.; Schingaro E., Scordari, F. *Eur. J. Org. Chem.* **2000**, 869; b) Herrmann, W.A.; Elison, M.; Fischer, J.; Kocher, C.; and Artus, G.R.J. *Angew. Chem. Int. Ed. Engl.*, **1995**, 34, 2371-2374.

3 Bowman, W.R.; Stephenson, P.T.; Young, A.R. *Tetrahedron*, **1996**, *52*, 11445-11462.

4 Procop, J.; Merica, R.; Glatz, F.; Veprek, S.; Klingan, F.R.; Ermann, W.A. *Non-Cryst. Solids*, **1996**, 34, 2368.

5 Grieco, P.A.; Finkhelhor, R. *J. Org. Chem.*, **1973**, 38, 2100-2101.

6 Cuvigny, T.; Julia, M. *J. Org. Chem.*, **1987**, 331, 121-138.

7 Takahashi, K. et al. Bull. Chem. Soc. Jpn.; 1972, 45, 1183-1191.

8 Harries et al. Inorg. Chem. Acta, 1978, 31, 233-235.

9 Padwa, A.; Ishida, M.; Cheryl, L.; Murphree, S.S. *J. Org. Chem.*, **1992**, 57, 1170-1178.
